# Risk factors for acute chest syndrome among children with sickle cell anemia hospitalized for vaso-occlusive crises

**DOI:** 10.1038/s41598-023-48527-1

**Published:** 2024-03-12

**Authors:** Faisal A. Alghamdi, Fawaz Al-Kasim, Forat Alshhada, Eatedal Ghareeb, Fauzia R. Azmet, Adel Almudaibigh, Lobna Baitalmal, Bedah Alnawfal, Rehab Alluqmani

**Affiliations:** 1 Department of Pediatric Hematology and Oncology, King Saud Medical City, Riyadh, Saudi Arabia; 2 Medical Specialized Center, Riyadh, Saudi Arabia

**Keywords:** Respiratory tract diseases, Predictive markers, Prognostic markers, Paediatrics, Paediatric research, Pain, Diseases, Haematological diseases, Sickle cell disease

## Abstract

Sickle cell anemia (SCA) is a globally prevalent inherited condition, with acute chest syndrome (ACS) being one of its most severe complications. ACS frequently leads to hospitalization, requires intensive care unit (ICU) admission, and can even result in death. This study aimed to discern the early indicators of impending ACS in children with SCA who were initially hospitalized due to painful vaso-occlusive crises (VOC). This was a retrospective, case‒control investigation of 120 patients aged 1–14 years seen at the King Saud Medical City in Riyadh, Saudi Arabia from January 2021 to December 2022. Patients were classified into cases and controls: those who developed and did not develop ACS during hospital stay, respectively. Demographic factors, laboratory results, vital and clinical signs, and treatment protocols were compared between these groups. The following were significant predictors of impending ACS: previous diagnosis of asthma, history of ACS, recent upper respiratory tract symptoms prior to admission, and need for a blood transfusion within the first 24 h of admission due to a drop in hemoglobin levels. Further regression analysis indicated that elevated steady-state mean corpuscular volume, leukocyte count, total bilirubin, and an increased absolute neutrophil count level 24 h after admission also foreshadowed impending ACS among patients admitted for VOC. The location of pain was also significant; the incidence of ACS was higher in patients with back pain, but lower in those with pain confined to the limbs. The ACS group had a longer average duration of hospital stay compared to those with VOC alone, (7.6 vs. 5.8 days). Among patients initially admitted for VOC, 15.7% were diagnosed with ACS. Most ACS cases were managed with transfusions and antibiotics, and nearly one-third of patients needed admission to an ICU or a high-dependency area.

## Introduction

Sickle cell anemia (SCA) is a prevalent genetic disorder in Saudi Arabia^1,2^ characterized by hemolytic anemia and abnormally shaped red blood cells^3^. However, the full prevalence of SCA has been historically underestimated due to a lack of neonatal screening^4^, but this has begun to change with the recent implementation of neonatal screening in 2023. In Saudi Arabia, the carrier status of sickle cell disorders ranges between 2 and 27%, with 1.4% of individuals in the eastern region of the country^4^. This high prevalence is linked to the substantial rate of consanguineous marriages, which reaches 52%^5^.

One of the most severe complications of SCA is acute chest syndrome (ACS), an acute illness characterized by new segmental pulmonary infiltrates associated with fever and/or various respiratory symptoms^1^. ACS accounts for 43% of pediatric SCA-related admissions to the intensive care unit (ICU). It is the leading cause of death in patients with SCA, underscoring the need for early diagnosis and intervention^1,2,6,7^.

A temporal relationship has been established between ACS and vaso-occlusive crises (VOCs) in SCA patients; nearly half of ACS cases are diagnosed 2–3 days after admission for an acute painful crisis^1,8^. A retrospective study showed that 33% of patients diagnosed with ACS sought medical advice for prodromal symptoms prior to the diagnosis of ACS^9^. A recent multicenter retrospective study in Italy revealed that almost 75% of children diagnosed with ACS were concurrently experiencing pain. In 32% of those cases, the severity of pain necessitated the use of morphine^10^. Elevated serum phospholipase A2 levels and a decline in platelet count greater than 10% have been identified as significant predictors for the development of ACS in adults with SCA^11^. Additionally, a retrospective case‒control study in 2020 demonstrated that asplenia, fever, reduced oxygen saturation, low hemoglobin, and leukocytosis are important risk factors for the onset of ACS among adult SCA patients^12^.

A variant of rapidly progressive ACS can quickly develop from mild hypoxemia to severe respiratory failure within 24 h, even if chest X-ray results are normal. This ACS variant can potentially advance to multiorgan failure and death; it is mainly found in adults but can also occur in children^1,13,14^.

Despite extensive research in SCA and ACS, the risk determinants of ACS in pediatric SCA patients admitted for VOC remains unknown. The current body of literature is limited, encompassing only a few adult studies^12,15^ and a single prospective pediatric study by Madhi et al., which identified laboratory metrics (e.g., reticulocyte and neutrophil counts) and clinical indicators (e.g., pain score and location) as potential harbingers of ACS. Another notable predictor is the variation in leukocyte count and CRP levels on the second day of admission^16^.

A significant gap still exists in our understanding of the risk factors for ACS among pediatric patients with SCA admitted due to VOC. To fill this gap, this study investigated these risk factors in a large cohort of patients through a retrospective chart review at one of Saudi Arabia's large pediatric hematology centers. We aim to enhance our understanding of these risk factors to improve patient care and outcomes for children with SCA admitted for VOC.

## Materials and methods

### Study design and context

This is a retrospective, case‒control study carried out at the Pediatric Hematology and Oncology Department of King Saud Medical City, a tertiary care hospital in Riyadh, Saudi Arabia.

### Study participants

The study included pediatric patients aged between 1 and 14 years diagnosed with SCA who were admitted due to VOCs within a two-year period from January 2021 to December 2022.

### Inclusion and exclusion criteria

Patients diagnosed with SCA (either HbSS or HbS-β0 thalassemia) and were admitted during the study period for VOC were eligible for the study. Exclusion criteria included having other SCA subtypes, being diagnosed with acute chest syndrome (ACS) on the first day of admission or being on a chronic transfusion program.

### Data collection

Clinical and laboratory data were obtained from electronic medical records. The following demographic and clinical information were collected: age, sex, SCA type, details of pain, history of asthma/ACS, respiratory examination findings, and oxygen saturation at admission. The laboratory data included complete blood count, bilirubin, and hemoglobin levels at routine clinic visits, on emergency room arrival, and 24 h post admission. The instances, timing, management, and outcomes of ACS were also documented.

### Definitions

SCA was defined as either sickle cell SS disease or S-beta zero thalassemia^17^. VOC was defined as hospitalization due to pain that necessitated parenteral narcotics. ACS was defined as any respiratory symptom, chest pain, or fever associated with a new lung infiltrate on chest X-ray^8^. A history of asthma was defined based on a pediatrician's report or a prescription history of asthma controller medication.

### Ethical considerations

This study was conducted in strict compliance with ethical principles, receiving approval from the Ethics Review Board at King Saud Medical City, Riyadh. Given the study's design, there was no direct interaction with patients or use of tissue samples, eliminating the need for parental or guardian informed consent for minors. In recognition of this, our Ethics Review Board granted a waiver for informed consent. All patient data were anonymized to ensure confidentiality, and we affirm that our methods align with relevant guidelines and regulations.

### Statistical analysis

Categorical variables are displayed as numbers and percentages. Continuous variables are depicted as the mean and standard deviation. The relationship between the development of ACS and demographic/clinical characteristics was assessed using chi-square tests and independent sample t tests. Univariate and multivariate logistic regression analyses were conducted to identify the predictors of ACS, and odds ratios and 95% confidence intervals were calculated. Paired t tests were employed to compare laboratory values at baseline, upon arrival at the emergency department, and 24 h after admission. Statistical significance was set at p < 0.05, and all analyses were performed using SPSS version 26.

## Results

This retrospective study, aimed to pinpoint risk factors for ACS among children with sickle cell anemia hospitalized for VOCs. The study involved 197 VOC admissions from 120 pediatric patients over 2 years at a tertiary hospital.

Regarding patient demographics (Table 1), 50.8% were between 8 and 14 years old, with a mean age of 7.33 ± 3.17 years. Notably, 55.8% were females, 93.4% were of Saudi ethnicity, and 74.1% had multiple hospital admissions in the studied period. Nearly half of the patients had an average of 1–2 hospital admissions per year due to VOC. Interestingly, the frequency of hospital admissions was not significantly correlated with the incidence of ACS post admission. Hemoglobin SS was the most frequently observed form (80%) of SCA, and 45.1% were taking hydroxyurea before admission. Regarding past medical histories, 16.8% had asthma, 24.9% had previously experienced ACS, and 14.7% had a recent upper respiratory tract infection (URTI).

**Table 1:** Relationship between ACS development among the demographic and clinical characteristics (n = 197).

Factor	All VOC admissions	ACS during VOC admission		P value^§^
	N (%) (n = 197)	Yes N (%) (n = 31)	No N (%) (n = 166)	
Age group				
1–7 years	97 (49.2%)	10 (32.3%)	87 (52.4%)	0.039**
8–14 years	100 (50.8%)	21 (67.7%)	79 (47.6%)	
Gender				
Male	87 (44.2%)	12 (38.7%)	75 (45.2%)	0.505
Female	110 (55.8%)	19 (61.3%)	91 (54.8%)	
Number of episodes for the patient during the study period				
Single	51 (25.9%)	08 (25.8%)	43 (25.9%)	0.991
Multiple	146 (74.1%)	23 (74.2%)	123 (74.1%)	
Number of VOC admissions per year for the patient:				
< 1 VOC admission per year	54 (27.4%)	06 (19.4%)	48 (28.9%)	0.436
1–2 VOC admission per year	98 (49.7%)	19 (61.3%)	79 (47.6%)	
3–4 VOC admission per year	36 (18.3%)	04 (12.9%)	32 (19.3%)	
> 4 VOC admission per year	9 (04.6%)	02 (06.5%)	07 (04.2%)	
Type of SCA				
Hemoglobin SS	152 (80.0%)	25 (83.3%)	127 (79.9%)	0.661
HbS/β0 Thalassemia	37 (19.5%)	05 (16.7%)	32 (20.1%)	
Previous diagnosis of asthma No (%)	33 (16.8%)	13 (41.9%)	20 (12.0%)	< 0.001**
Previous acute chest syndrome	49 (24.9%)	13 (41.9%)	36 (21.7%)	0.017**
Previous PICU admission for ACS	24 (12.2%)	05 (16.1%)	19 (11.4%)	0.464
Previous history of splenectomy	25 (12.7%)	03 (09.7%)	22 (13.3%)	0.772
Recent history of URTI within one week of visit? ^(n=95)^	29 (14.7%)	11 (64.7%)	18 (23.1%)	0.001**
On hydroxyurea before admission	89 (45.1%)	18 (58.1%)	71 (42.8%)	0.116
Symptoms upon presentation to ER^†^				
Cough	20 (10.2%)	05 (16.1%)	15 (09.0%)	0.326
SOB	06 (3.1%)	01 (03.2%)	05 (03.0%)	1.000
Fever	65 (32.9%)	07 (22.6%)	58 (34.9%)	0.179
Duration of pain before visit				
1 day	117 (59.4%)	16 (51.6%)	101 (60.8%)	0.287
2 days	48 (24.4%)	11 (35.5%)	37 22.3%)	
3 days	32 (16.2%)	04 (12.9%)	28 (16.9%)	
Pain severity				
Mild	76 (38.6%)	10 (32.3%)	66 (40.7%)	0.070
Moderate	92 (46.7%)	20 (64.5%)	72 (44.4%)	
Severe	25 (12.7%)	01 (03.2%)	24 (14.8%)	
Site of pain†				
Back	104 (52.7%)	23 (74.2%)	81 (48.8%)	0.009**
Only upper and/or lower limb	51(25.8%)	03 (09.7%)	48 (28.9%)	0.025**
Chest/Sternum	32(16.2%)	07 (22.6%)	25 (15.1%)	0.297
Upper limb	57(28.9%)	04 (12.9%)	53 31.9%)	0.032**
Lower limb	104(52.7%)	14 (45.2%)	90 (54.2%)	0.354
Abdomen	52(26.3%)	08 (25.8%)	44 (26.5%)	0.935
Diffuse	19(09.6%)	02 (06.5%)	17 (10.2%)	0.512

*SCA* sickle cell anemia, *VOC* vaso-occlusive pain crises, *ACS* acute chest syndrome.

^†^Some patients have more than one finding.

^§^P value was calculated using the chi-square test.

**Significant at the p < 0.05 level.

Upon admission, 98% of patients had normal chest X-ray findings. Moderate pain was reported in 46.7% of cases, and 44.2% underwent chest x-rays in the emergency room (ER). The following factors had a significantly increased risk for ACS: older age (p = 0.039), a history of asthma (p < 0.001), prior ACS (p = 0.017), recent URTI (p = 0.001), and back pain (p = 0.009). On the other hand, isolated limb pain was linked with a decreased risk (p = 0.025, p = 0.032). Administration of hydroxyurea before admission did not significantly alter the risk of developing ACS (p = 0.116) (Table 1).

In the ER (Table 2), decreased mean oxygen saturation was linked with a higher risk of ACS during hospitalization (p = 0.004). Fever ≥ 38 °C in the ER was seen in 17.3% of cases, while 3% initially had an oxygen saturation of < 94% in the ER.

**Table 2:** Initial vital signs at the ED in relation to ACS development during VOC admission ^(n = 197)^.

Vital signs	All VOC admissions	ACS during VOC admission		P value^§^
	Mean ± SD	Yes	No	
		Mean ± SD	Mean ± SD	
Axillary body temperature, C^‡^	37.2 ± 0.75	37.2 ± 0.73	37.2 ± 0.75	0.094
Heart rate, beats/minutes^‡^	121.7 ± 20.5	127.8 ± 18.6	121.1 ± 20.9	0.351
Respiratory rate, breaths/minute^‡^	26.8 ± 3.78	26.7 ± 3.01	26.8 ± 3.91	0.876
Oxygen saturation (%)^‡^	97.4 ± 2.23	96.3 ± 2.30	97.6 ± 2.16	0.004**
Documented Fever 38 C or more in ED N (%)^§^	34 (17.3%)	05 (16.1%)	29 (17.5%)	0.856
Oxygen saturation Less Than 94 in ED N (%)^§^	06 (03.0%)	03 (09.7%)	03 (01.8%)	0.051

*SCA* sickle cell anemia, *VOC* vaso-occlusive pain crises, *ACS* acute chest syndrome, *ED* emergency department.

^§^P value was calculated using the chi-square test.

^‡^P value was calculated using an independent sample t test.

**Significant at the p < 0.05 level.

**Figure 1:**
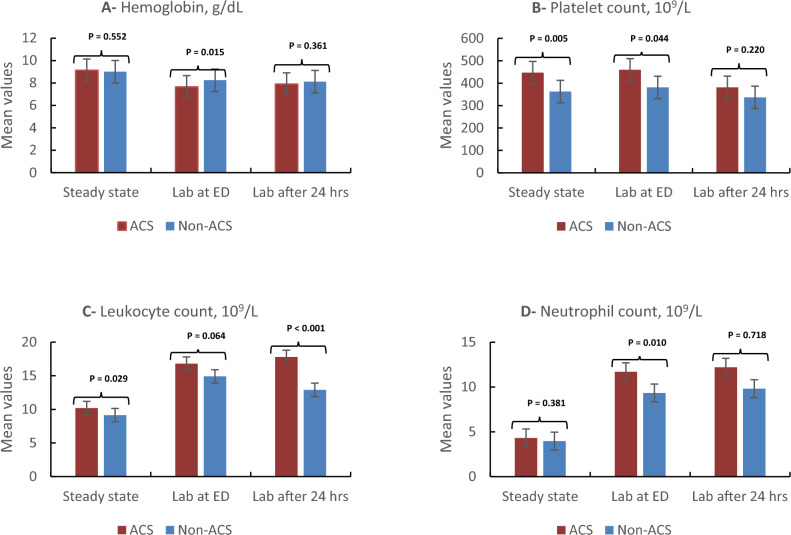
Comparison of laboratory values at steady state, emergency and at 24 h of VOC admission based on the development of ACS. Comparison of laboratory values for patients with acute chest syndrome (ACS) after vaso-occlusive crisis (VOC) admission versus the VOC-only group. (**a**) Upon arrival at the emergency department, hemoglobin levels were significantly lower in the ACS group than in the VOC-only group. (**b**) Platelet count was significantly higher in the ACS group at both steady state and upon arrival at the emergency department. (**c**) Leukocyte count was significantly higher in the ACS group at steady state and after 24 h. (**d**) Neutrophil count was significantly higher in the ACS group only upon arrival at the emergency department.

The following laboratory findings were associated with an increased risk for post-admission ACS: higher leukocyte count, platelet count, mean corpuscular volume, and total bilirubin at baseline (p < 0.05) (Table 3). Moreover, elevated neutrophil count, mean corpuscular volume, and platelet count in the emergency department (ED) were also associated with ACS occurrence (p < 0.05). Lower hemoglobin levels in the ED and a larger drop from baseline were also significantly associated with ACS (p ≤ 0.019). Additionally, higher leukocyte counts and total bilirubin 24 h after admission correlated with ACS risk (p < 0.05) (Fig. 1).

**Table 3:** Laboratory values at baseline, upon ED arrival, and after 24 h of admission in relation to ACS development during admission (n = 197).

	All VOC admissions	ACS during VOC admission		P value^§^
	(No.197)	Yes (No.31)	No (No.166)	
	Mean ± SD	Mean ± SD	Mean ± SD	
Laboratory values at steady state				
Hemoglobin S (%)	80.7 ± 9.12	82.5 ± 9.65	80.4 ± 9.02	0.252
Hemoglobin F (%)	12.3 ± 6.72	10.6 ± 6.06	12.6 ± 6.80	0.149
Hemoglobin A2 (%)	3.87 ± 0.94	3.62 ± 0.65	3.91 ± 0.98	0.130
Leucocyte count 10^9^/L	9.30 ± 2.43	10.2 ± 2.12	9.14 ± 2.45	0.029**
Neutrophil count 10^9^/L	4.02 ± 1.96	4.32 ± 1.72	3.97 ± 1.99	0.381
Hemoglobin, g/dL	9.03 ± 1.05	9.14 ± 1.08	9.01 ± 1.05	0.552
Platelet count 10^9^/L	375.7 ± 153.2	446.9 ± 131.0	362.6 ± 153.7	0.005**
Mean corpuscular volume, fl	80.8 ± 10.5	85.6 ± 10.2	79.9 ± 10.4	0.005**
Reticulocyte percentage (%)	6.51 ± 2.92	7.31 ± 3.44	6.35 ± 2.79	0.094
Total bilirubin level, µmol/L	21.7 ± 11.4	26.5 ± 16.8	20.8 ± 9.84	0.011**
Laboratory values at presentation to ED				
Leucocyte count 10^9^/L	15.2 ± 4.99	16.8 ± 5.53	14.9 ± 4.96	0.064
Neutrophil count 10^9^/L	9.75 ± 4.34	11.7 ± 4.67	9.34 ± 4.25	0.010**
Hemoglobin, g/dL	8.17 ± 1.24	7.66 ± 1.44	8.25 ± 1.18	0.015**
Platelet count 10^9^/L	397.8 ± 197.3	459.5 ± 268.6	381.1 ± 181.5	0.044**
Mean corpuscular volume, fl	80.3 ± 9.89	83.5 ± 10.2	79.6 ± 9.77	0.045**
Reticulocyte percentage (%)	9.74 ± 4.11	10.7 ± 4.42	9.53 ± 4.03	0.172
Total bilirubin level, µmol/L	37.8 ± 35.0	41.3 ± 17.2	37.0 ± 37.2	0.541
Uric acid	221.9 ± 70.7	203.1 ± 70.6	225.6 ± 70.4	0.125
ESR		47.9 ± 38.4	43.8 ± 32.5	0.584
CRP	69.7 ± 83.9	80.4 ± 111.0	66.7 ± 75.3	0.522
Laboratory values after 24 h of admission				
Leucocyte count 10^9^/L	13.7 ± 5.56	17.8 ± 5.57	12.9 ± 5.22	< 0.001**
Neutrophil count 10^9^/L	10.2 ± 30.6	12.2 ± 4.75	9.82 ± 3.4	0.718
Hemoglobin, g/dL	8.09 ± 1.16	7.91 ± 1.49	8.12 ± 1.09	0.361
Platelet count 10^9^/L	343.9 ± 184.9	381.3 ± 206.2	336.7 ± 180.5	0.220
Reticulocyte percentage (%)	9.59 ± 4.62	9.44 ± 4.62	9.62 ± 4.64	0.854
Total bilirubin level, µmol/L	34.5 ± 28.7	47.7 ± 22.9	31.9 ± 29.1	0.006**

*SCA* sickle cell anemia, *VOC* vaso-occlusive pain crises, *ACS* acute chest syndrome, *ED* emergency department.

^§^P value was calculated using an independent sample t test.

**Significant at the p < 0.05 level.

**Figure 2:**
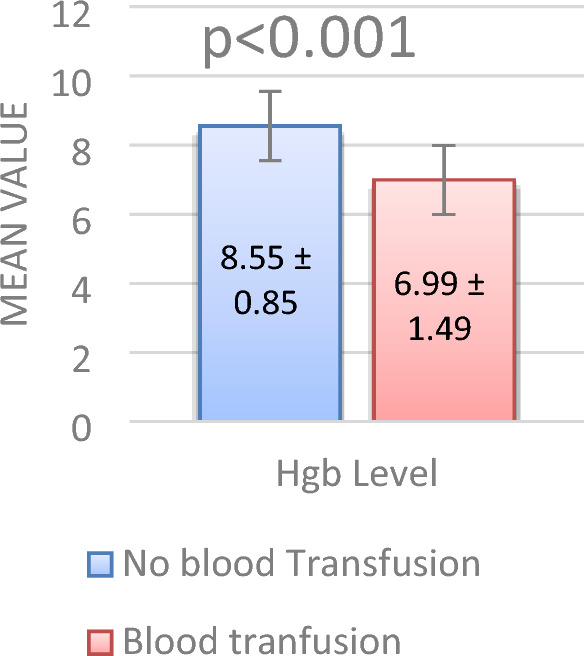
Association between blood transfusion in the first 24 h and hemoglobin level in the ED. Patients who had blood transfusions had a lower mean hemoglobin level in the ER (p < 0.001).

VOC was initially managed by maintenance intravenous fluids (49.7%), morphine (83.2%; with 36% receiving morphine infusions), and incentive spirometry within the first 24 h (12.2%) (Table 4). Interestingly, 25% of patients received blood transfusions within the first 24 h, and this was linked with a higher ACS risk (p < 0.001). On the other hand, codeine use was associated with a lower ACS risk (p < 0.001) (Fig. 2).

**Table 4:** Relationship between initial management received for VOC admissions and ACS during VOC admission (n = 197).

Factor	All VOC admissions	ACS during VOC admission		P value^§^
	N (%) (n = 197)	Yes	No	
		N (%) (n = 31)	N (%) (n = 166)	
IV fluid (IVF)				
Maintenance	98 (49.7%)	14 (45.2%)	84 (51.2%)	0.729
More than maintenance	80 (40.6%)	15 (48.4%)	65 (39.6%)	
Less than maintenance or no IVF	19 (09.6%)	02 (06.5%)	17 (10.2%)	
Pain management^†^				
Morphine infusion	72 (36.5%)	16 (51.6%)	56 (33.7%)	0.058
Morphine intermittent doses (Regular)	64 (32.4%)	11 (35.5%)	53 (31.9%)	0.698
Codeine	103 (52.2%)	07 (22.6%)	96 (57.8%)	< 0.001**
Incentive spirometry 1st 24 h of admission	24 (12.2%)	07 (22.6%)	17 (10.2%)	0.054
Blood transfusion during first day of VOC admission before onset of ACS	49 (24.8%)	16 (51.6%)	33 (19.9%)	< 0.001**

*SCA* sickle cell anemia, *VOC* vaso-occlusive pain crises, *ACS* acute chest syndrome.

^†^Some patients have more than one finding.

^§^P value was calculated using the chi-square test.

**Significant at the p < 0.05 level.

The development of ACS during hospitalization was significantly associated with a considerable drop in hemoglobin levels in the ED compared to baseline steady-state levels, as well as a significant increase in leukocyte count difference, measured at the ED and 24 h later (p < 0.001) (Table 5).

**Table 5:** Difference in laboratory values at baseline steady state, upon arrival at the ED, and after 24 h of admission in relation to ACS diagnosis (n = 197).

Variable	All VOC admissions	ACS during VOC admission		P value^§^
	(No.197)	Yes (No.31)	No (No.166)	
Laboratory value at arrival to ED in comparison to steady stat value:				
WBC 10^9^/L	+ 5.9	+ 6	+ 5	0.621
HB g/dl	− 0.86	− 1.47	− 0.75	0.006**
PLT 10^9^/L	+ 22.1	+ 23	+ 21	0.978
ANC 10^9^/L	5.74	+ 7	+ 5	0.073
Laboratory value after 24 h of admission in comparison to ED value:				
WBC 10^9^/L	-1.5	+ 1	− 2	< 0.001**
HB g/dl	+ 0.07	+ 0.24	− 0.13	0.080
PLT 10^9^/L	− 49.6	− 78	− 46	0.158
ANC 10^9^/L	+ 0.45	+ 0.5	+ 0.48	0.856

*WBC* Leukocyte count, *HB* hemoglobin g/dl, *PLT* platelet count, *ANC* absolute neutrophil count, *SCA* sickle cell anemia, *VOC* vaso-occlusive pain crises, *ACS* acute chest syndrome, *ED* emergency department.

^§^P value was calculated using an independent sample t test.

**Significant at the p < 0.05 level.

+ increased by, − decreased by.

**Figure 3:**
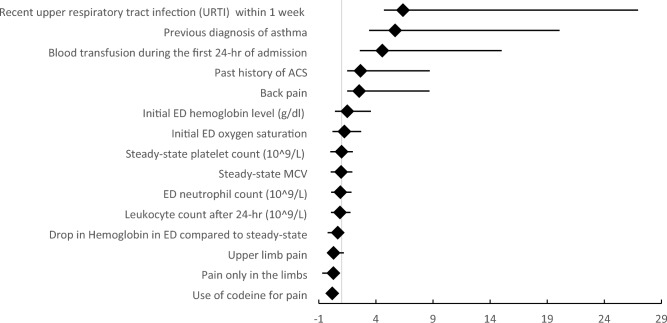
Forest plot for the multivariate regression of significant risk factors for ACS admission. *ED* emergency department, *ACS* acute chest syndrome, Forest plot for predictors of acute chest syndrome during vaso-occlusive crisis admission based on multivariate adjusted odds ratio (AOR).

Regression analysis revealed the following factors significantly associated with the development of ACS during VOC in children with SCA: older age, prior diagnosis of ACS or asthma, recent history of URTI, back pain, receiving a simple blood transfusion in the first 24 h, lower initial O2 saturation and hemoglobin levels at ED, and a decrease in hemoglobin values compared to baseline (Table 6). Codeine use for pain management, however, reduced the ACS risk (Fig. 3).

**Table 6:** Univariate and multivariate regression analysis to determine the influence predictors of ACS development during admission.

Factor	OR (95% CI)	P value	AOR (95% CI)	P value
Age group				
1–7 years	Ref			
8–14 years	2.313 (1.026–5.211)	0.043**	2.245 (0.965–5.227)	0.061
Previous diagnosis of asthma	5.272 (2.247–12.37)	< 0.001**	5.683 (2.284–14.44)	< 0.001**
Previous acute chest syndrome	2.608 (1.168–5.823)	0.019**	2.651 (1.159–6.066)	0.021**
Recent history of URTI within 1 week of visit? ^(n=95)^	6.111 (1.983–18.83)	0.002**	6.371 (1.672–20.58)	0.002**
Back pain	3.017 (1.277–7.131)	0.012**	2.537 (1.046–6.157)	0.040**
Site of pain: Upper limb	0.316 (0.105–0.949)	0.040**	0.290 (0.092–0.914)	0.035**
Only upper and/or lower limb pain	0.263 (0.076–0.907)	0.035**	0.279 (0.080–0.978)	0.046**
Pain management: Codeine	0.213 (0.087–0.521)	0.001**	0.180 (0.071–0.457)	< 0.001**
Simple blood transfusion in 1st 24 h	4.299 (1.930–9.576)	< 0.001**	4.563 (1.969–10.45)	< 0.001**
Initial O_2_ saturation	1.249 (1.059–1.473)	0.008**	1.246 (1.053–1.474)	0.010**
Steady-state laboratory value				
Leucocyte count 10^9^/L	0.847 (0.727–0.986)	0.032**	0.863 (0.737–1.009)	0.065
Platelet count 10^9^/L	0.997 (0.994–0.999)	0.007**	0.996 (0.994–0.999)	0.009**
Mean corpuscular volume, fl	0.947 (0.911–0.985)	0.007**	0.953 (0.915–0.991)	0.017**
Total bilirubin level, µmol/L	0.965 (0.937–0.993)	0.016**	0.971 (0.941–1.002)	0.067
Emergency laboratory value				
Neutrophil count 10^9^/L	0.889 (0.810–0.975)	0.013**	0.890 (0.808–0.981)	0.018**
Hemoglobin, g/dL	1.423 (1.060–1.910)	0.019**	1.496 (1.082–2.067)	0.015**
Platelet count 10^9^/L	0.998 (0.997–1.000)	0.055	0.998 (0.996–1.000)	0.061
Mean corpuscular volume, fl	0.960 (0.922–0.999)	0.047**	0.961 (0.923–1.001)	0.055
24 h post-admission laboratory value				
Leucocyte count	0.862 (0.803–0.925)	< 0.001**	0.859 (0.797–0.925)	< 0.001**
Total bilirubin level, µmol/L	0.984 (0.970–0.999)	0.038**	0.987 (0.972–1.001)	0.078
Laboratory value difference:				
Hemoglobin drops in ED versus steady state	0.687 (0.522–0.905)	0.007**	0.661 (0.494–0.886)	0.006**
Leucocyte increase after 24 h of admission compared to ED	0.833 (0.753–0.922)	< 0.001**	0.845 (0.762–0.937)	0.001**

Adjusted for age, gender, nationality, and BMI.

*OR* odds ratio, *AOR* adjusted odds ratio, *CI* confidence interval.

*SCA* sickle cell anemia, *VOC* vaso-occlusive pain crises, *ACS* acute chest syndrome, *ED* emergency department.

**Significant at the p < 0.05 level.

On average, ACS was typically diagnosed 87.5 ± 174.0 h after admission, presenting with fever (93.5%) and respiratory symptoms (93.5%). Chest x-rays revealed new infiltrates in all episodes, most frequently in the right lower lobe (51.6%), followed by the left upper lobe (25.8%).

Antimicrobial management included ceftriaxone (96.8%), azithromycin (90.3%), and vancomycin (41.9%). Simple top-up red blood cell transfusions were used in 90.3% of cases, while exchange transfusion was performed in 6.5%. Ventolin nebulization was done in 80.6% of patients. Moreover, 29.0% of ACS patients needed admission to pediatric intensive care or high-dependency units.

Hospital stay was prolonged when ACS developed, averaging 5.82 days for VOC alone and 7.65 days when ACS occurred (p = 0.002). No in-hospital mortality was observed in our sample.

## Discussions

Sickle cell disorder is highly prevalent in Saudi Arabia^4^. Among SCA patients, ACS is a leading cause of mortality and ICU admission^2,18,19^. VOCs are the most common reason for hospitalization among SCA patients^1,2,12,18,19^. Previous research has shown that approximately 13%–20% of patients will go on to develop ACS following admission for a VOC in the initial 3 days over an indolent clinical course^12,13,16,20^.

This study aimed to identify risk factors that could predict the development of ACS among pediatric patients with SCA, in the hopes of helping clinicians focus monitoring and care efforts on higher-risk patient groups. Among the SCA children admitted to our hospital for VOC, 15.7% developed ACS after an average of 87 h (3.6 days) following admission**.** Our findings revealed that a substantial percentage of ACS cases emerged following admissions predominantly tied to crises such as pain. The data set in the study was well-positioned to explore the risk factors for post-admission ACS progression.

A previous study by Bellet et al. found that, among pediatric SCA patients hospitalized for VOC, those who performed incentive spirometry had an 87% lower relative risk of developing ACS compared to those who did not^21^. In the present study, incentive spirometry was notably underutilized, used in only 12.2% of patients. This underuse is striking, especially considering its proven efficacy. Similarly, a recent Italian study reported spirometry being used in only 16% of ACS patients^10^. Other studies have shown the benefits of a multidisciplinary preventive approach, which reduced the risk of ACS during admissions for VOC from 25 to 12%^22^. Since there are evidence-based preventive that can lower the incidence of ACS, the aim now should be to identify which patients are at highest risk.

Patients aged 8–14 years exhibited a stronger association with ACS during admission than younger children. This finding differed from the observational study of Madhi et al., potentially due to dissimilar age groupings between the two^16^. However, our observations aligned with a large multicenter study by Vichinsky et al., which reported a median age of 5.4–14.6 years at the first ACS episode in patients aged 0–9 and 10–19 years, respectively^7^. Notably, our study suggests that gender does not influence post-admission ACS risk on its own within our population.

In the present study, a history of asthma and prior ACS episodes predicted a higher risk of post-VOC admission ACS. These findings align with multiple prospective and retrospective studies suggesting that pediatric SCA patients with asthma have a greater incidence of ACS versus those without asthma^23,24^. The proinflammatory effects of asthma may contribute to this relationship^24^.

We found that URTI symptoms in the week prior to VOC were linked to a markedly higher risk of post-admission ACS (p = 0.001). Other studies have also noted this relationship between recent URTI and ACS^9,20,25,26^. URTI may induce pain episodes that promote ACS or directly cause ACS through indolent viral infection exacerbated by pain.

In our analysis, no statistically significant difference was observed between patients with sickle cell SS disease and those with sickle S-beta-zero thalassemia in terms of risk for developing ACS during admission. Notably, our study only included these two specific types of sickle cell disease and excluded other variants. Given this limited scope, our finding of no discernable risk difference between SS disease and S-beta-zero thalassemia for ACS occurrence is reasonable based on the sample size of each group.

Majority (74.2%) of patients in the ACS group presented with back pain upon admission, versus 48% in the VOC-only group (p = 0.009). In contrast, only a minority (9.7%) of the ACS group reported exclusive limb pain versus 28.9% in those without ACS (p = 0.025), suggesting that isolated extremity pain is associated with a lower risk of post-admission ACS, while back pain is associated with an increased risk. These findings are in agreement with the observational study of Madhi et al. and pain-induced hypoventilation theories linking pulmonary dysfunction to ACS development^16,27^. In agreement with previous research, we found that decreased oxygen saturation upon arrival at the ED was associated with a greater risk of developing ACS^12,16,28^.

In contrast to other research^12,16^, we found no significant differences between the ACS and VOC-only groups regarding other initial exam findings in the ED, such as reported pain scores. While ACS patients tended to report more severe pain, this difference was not statistically significant, likely due to the limitations of retrospective pain documentation review. As previously reported in the literature, we found no statistically significant difference in ACS risk during VOC admission based on intravenous hydration volume^29,30^.

ACS occurred significantly less among patients receiving codeine during admission. However, this may reflect an association rather than a causation, because codeine was used for mild to moderate pain at our study hospital. Morphine infusions had a trend toward increased ACS risk (p = 0.058), consistent with other studies linking morphine to hypoventilation-driven ACS due to its sedation effect and possibly reflecting more severe pain^16^.

Patients receiving red blood cell transfusions within the first 24 h of VOC admission were at a significantly higher risk of developing ACS during hospitalization (p < 0.001). These transfusions correlated with a decrease in hemoglobin (p = 0.001). Transfused patients also needed additional transfusions after the diagnosis of ACS. Thus, while transfusions cannot be proven as the direct cause of ACS, these were not protective. On the other hand, Madhi et al. found that 17.9% of VOC-only children received early transfusions without later developing ACS during admission^16^. A Cochrane analysis in 2020 found no clear evidence for transfusions in ACS due to a lack of randomized data^31,32^. It remains uncertain whether early transfusion during VOC or early in an ACS course improves outcomes. Prospective trials are needed to compare transfusions to supportive care approaches to better guide clinical management, especially during episodes of VOC.

In agreement with previous research^2,12,14,16,33^, a higher leukocyte count, platelet count, mean corpuscular volume, and total bilirubin at baseline were all associated with an increased ACS risk during VOC admission. Elevations in these markers could reflect a greater degree of underlying hemolysis and disease severity. Although not statistically significant, lower baseline hemoglobin F levels and higher reticulocyte percentages in the ACS group correlated with prior findings linking lower hemoglobin F and higher ACS risk^2^.

Upon arrival at the ED, significantly lower hemoglobin levels were seen in patients who eventually developed ACS. These patients, compared to the VOC-only group, had a greater decrease in hemoglobin from steady-state levels (1.47 vs. 0.75 g/dL; p = 0.006). This may explain the higher transfusion rates seen in ACS patients before onset, as well as challenge the benefits of transfusion for preventing ACS during VOC episodes. Early changes in hemoglobin could help anticipate which patients may develop ACS and guide preventive actions, although the exact pathophysiological cause requires further research.

Consistent with other pediatric and adult studies^12,16^, higher white blood cell counts at triage, specifically neutrophils and platelets, positively correlated with ACS risk during admission. These are known adhesion molecules that can promote endothelial adhesion and the pathogenesis of sickle cell disease^34^.

Leukocyte count 24 h post admission remained significantly elevated in the ACS group compared to emergency levels, but dropped in the VOC-only group. This predictive pattern of leukocyte trajectory was also seen in the prospective study of Madhi et al.^16^.

In agreement with previous studies, further regression analysis showed that a history of asthma, previous ACS, recent URTI within one week of presentation, and back pain at presentation were all independent risk factors for post-admission ACS^7,16,35–37^.

Multivariate regression revealed that simple blood transfusion within the first 24 h of admission again served as an independent risk factor for ACS. On the other hand, pain localized only to the extremities significantly reduced the risk of ACS during VOC admission. Similar findings were observed by Madhi et al.^16^.

Similar to previous studies^16,33^, higher platelet count and mean corpuscular volume at steady state were also independent risk factors for ACS during admission in our multivariate regression analysis. Lower hemoglobin and higher neutrophil count upon presentation at the ED were confirmed as predictors of ACS in both univariate and multivariate analyses.

In the assessment of laboratory values at three time points a higher leukocyte count upon presentation at the ED that did not improve by 24 h after admission was a significant independent predictor of risk for ACS. This is in agreement with the study of Madhi et al.^16^. Moreover, a drop in hemoglobin level from baseline to ER presentation was also an independent predictor based on multivariate regression analysis.

In this study, 15.7% (31/197 episodes) of VOC admissions were complicated by the development of ACS, which occurred at a median of 3.6 days after admission. This is comparable to the findings of Madhi et al. that 19% (35/176) of children admitted for VOC developed ACS at a median of 2–3 days after admission. The observed rate of ACS and its timing of onset align with these prior studies^7,16,38^.

The definition of ACS in this study was based on the 2015 guidelines by Howard et al.^19^. All ACS patients had infiltrates on chest X-ray, with the right lower lobe most commonly involved, followed by the left upper lobe, similar to previous reports^39,40^. Majority of ACS patients exhibited fever and respiratory symptoms. Regarding therapeutic approaches, simple top-up transfusions were done in 90% of ACS patients in this study, while only 6% underwent exchange transfusions. These findings suggest that the more frequent use of simple transfusions leads to a lower occurrence of exchange transfusions. This differs with a recent study by V. Munaretto et al., wherein simple transfusions were done in 72% of cases, while exchange transfusions were performed in 15%^10^. In our study, antibiotics, most frequently ceftriaxone and azithromycin, were administered in almost all patients. Notably, vancomycin was used in 42% of patients, which is significantly higher compared to other studies^10^. This increased usage could be because, in this study, patients developed ACS after their admission rather than upon arrival at the ED, as observed in other studies^10^. The NACSSG study found that 13% of pediatric and adult ACS patients required noninvasive ventilation^7^, but this was not assessed in our study. However, 29% of our ACS cohort required pediatric intensive care or high-dependency admission. Furthermore, no cerebrovascular events or mortalities occurred among the patients in our study population. Similar to other studies^7,16^, we found that the duration of hospital admission was significantly longer in the ACS group compared to the VOC-only group (7.65 vs. 5.8 days; p = 0.004).

**Figure 4:**
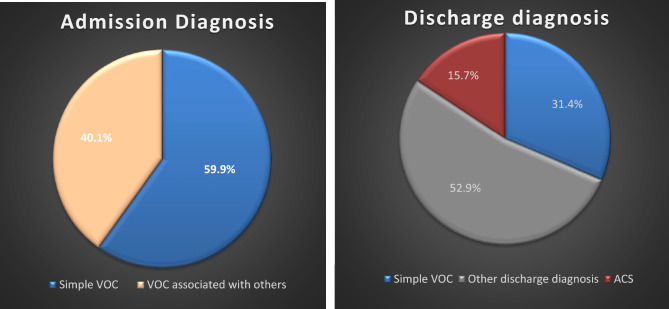
VOC is a diagnosis of exclusion. Distribution of diagnoses at admission and discharge. At admission, 59.9% of patients were diagnosed with a simple vaso-occlusive crisis (VOC), while 40.1% had VOC in conjunction with other diagnoses. Upon discharge, only 31.4% of patients maintained a diagnosis of simple VOC, while the remaining 69.6% had different discharge diagnoses.

Previous studies have observed that episodes of pain can potentially indicate various underlying conditions, including osteomyelitis^41^, stroke^42^, splenic sequestration^43^, and, notably, ACS, as seen in our study. To investigate this, we conducted a comparison of the admission and discharge diagnoses of patients (Fig. 4). Interestingly, 59.9% of the patients were initially admitted with a simple diagnosis of VOC, but only 31% of patients retained the diagnosis of simple VOC upon discharge. Most patients were discharged with different diagnoses, suggesting that VOC is indeed a diagnosis of exclusion. This underscores the critical importance of maintaining a high level of suspicion, even in seemingly uncomplicated pain crises.

Our study offers significant insights into predicting ACS among pediatric SCA patients admitted with VOC. Nevertheless, it is crucial to recognize certain constraints that, open avenues for additional research. Our research was confined to a single center and utilized retrospective data from a relatively small sample size. This limitation might curtail the broader applicability of our findings, especially when considering diverse patient populations in other settings.

An important goal for future research is to confirm these identified risk profiles through rigorous investigation, which could serve as the basis for developing a risk stratification tool.

Despite these limitations, we hope that our findings can encourage and support further research aimed at the early identification of at-risk pediatric patients with SCA. By targeting modifiable risk factors through prospective validation, we can ultimately improve outcomes for children affected by this disease.

## Conclusions

This retrospective study identified several potential risk factors for developing ACS during admission for VOC in pediatric SCA patients. Older age, history of asthma or prior ACS, back pain at presentation, blood transfusion in the first 24 h, recent URTI, higher baseline and presentation platelet count, leukocytosis, decreasing hemoglobin, and persistently elevated leukocyte count at 24 h were all associated with an increased risk of ACS. In contrast, pain confined to the extremities was associated with reduced risk. These indicators may help identify high-risk patients who could benefit from closer monitoring or from preventive measures during admissions for pain crises. Further prospective research is needed to validate these early predictors of impending ACS in diverse pediatric sickle cell populations. Refining risk assessment could ultimately lead to improved outcomes for this vulnerable group of patients.

## Data Availability

The raw data generated and analyzed during this study contain protected health information and are therefore not publicly available. Deidentified data may be available from the corresponding author upon reasonable request, pending approval from the ethical review board at King Saud Medical City Research Center. Any data shared will be fully anonymized to protect patient confidentiality and privacy in accordance with regulations.

## References

[1] Jain S, Bakshi N, Krishnamurti L (2017). Acute chest syndrome in children with sickle cell disease. Pediatr. Allergy Immunol. Pulmonol..

[2] Paul RN (2011). Acute chest syndrome: Sickle cell disease. Eur. J. Haematol..

[3] Lonergan GJ, Cline DB, Abbondanzo SL (2001). Sickle cell anemia. RadioGraphics.

[4] Jastaniah W (2011). Epidemiology of sickle cell disease in Saudi Arabia. Ann. Saudi Med..

[5] Al-Abdulkareem AA, Ballal SG (1998). Consanguineous marriage in an urban area of Saudi Arabia: Rates and adverse health effects on the offspring. J. Community Health.

[6] Bartram JL (2010). Outcome of children with sickle cell disease admitted to intensive care—A single institution experience. Br. J. Haematol..

[7] Vichinsky EP (2000). Causes and outcomes of the acute chest syndrome in sickle cell disease. N. Engl. J. Med..

[8] Styles LA (1996). Phospholipase A2 levels in acute chest syndrome of sickle cell disease. Blood.

[9] Creary SE, Krishnamurti L (2014). Prodromal illness before acute chest syndrome in pediatric patients with sickle cell disease. J. Pediatr. Hematol. Oncol..

[10] Munaretto V (2023). Acute chest syndrome in children with sickle cell disease: Data from a national AIEOP cohort identify priority areas of intervention in a hub-and-spoke system. Br. J. Haematol..

[11] Alhandalous CH (2015). Platelets decline during vaso-occlusive crisis as a predictor of acute chest syndrome in sickle cell disease. Am. J. Hematol..

[12] Alkindi S (2020). Predictors of impending acute chest syndrome in patients with sickle cell anaemia. Sci. Rep..

[13] Chaturvedi S (2016). Rapidly progressive acute chest syndrome in individuals with sickle cell anemia: A distinct acute chest syndrome phenotype. Am. J. Hematol..

[14] Yawn BP (2014). Management of sickle cell disease: Summary of the 2014 evidence-based report by expert panel members. JAMA.

[15] Bartolucci P (2016). Score predicting acute chest syndrome during vaso-occlusive crises in adult sickle-cell disease patients. EBiomedicine.

[16] Madhi F (2019). Identification of clinical and laboratory parameters associated with the development of acute chest syndrome during vaso-occlusive episodes in children with sickle cell disease: A preliminary step before assessing specific and early treatment strategies. J. Clin. Med..

[17] Yawn BP, John-Sowah J (2015). Management of sickle cell disease: Recommendations from the 2014 expert panel report. Am. Fam. Physician.

[18] Abd Elmoneim AA (2019). Causes of hospitalization in sickle cell diseased children in western region of Saudi Arabia. A single center study. Saudi Med. J..

[19] Howard J (2015). Guideline on the management of acute chest syndrome in sickle cell disease. Br. J. Haematol..

[20] Chang TP (2013). Clinical factors and incidence of acute chest syndrome or pneumonia among children with sickle cell disease presenting with a fever: A 17-year review. Pediatr. Emerg. Care.

[21] Bellet PS (1995). Incentive spirometry to prevent acute pulmonary complications in sickle cell diseases. N. Engl. J. Med..

[22] Reagan MM, DeBaun MR, Frei-Jones MJ (2011). Multi-modal intervention for the inpatient management of sickle cell pain significantly decreases the rate of acute chest syndrome. Pediatr. Blood Cancer.

[23] Boyd JH (2006). Asthma is associated with acute chest syndrome and pain in children with sickle cell anemia. Blood.

[24] Andemariam B (2015). The sickle cell mouse lung: Proinflammatory and primed for allergic inflammation. Transl. Res..

[25] George A (2011). The impact of the 2009 H1N1 influenza pandemic on pediatric patients with sickle cell disease. Pediatr. Blood Cancer.

[26] Strouse JJ (2010). Severe pandemic H1N1 and seasonal influenza in children and young adults with sickle cell disease. Blood.

[27] Gelfand MJ (1993). Simultaneous occurrence of rib infarction and pulmonary infiltrates in sickle cell disease patients with acute chest syndrome. J. Nucl. Med..

[28] Crabtree EA (2011). Improving care for children with sickle cell disease/acute chest syndrome. Pediatrics.

[29] Ojo AS (2022). Intravenous fluid administration and the risk of adverse outcomes in sickle cell disease patients hospitalized for vaso-occlusive crisis. J. Hematol..

[30] Gaut D (2020). Outcomes related to intravenous fluid administration in sickle cell patients during vaso-occlusive crisis. Ann. Hematol..

[31] Dolatkhah R, Dastgiri S (2020). Blood transfusions for treating acute chest syndrome in people with sickle cell disease. Cochrane Database Syst. Rev..

[32] StatPearls. 2023.

[33] Yousef AA (2022). Predictors of recurrent acute chest syndrome in pediatric sickle cell disease: A retrospective case-control study. Children (Basel).

[34] Telen MJ (2007). Role of adhesion molecules and vascular endothelium in the pathogenesis of sickle cell disease. Hematol. Am. Soc. Hematol. Educ. Program.

[35] Glassberg JA (2012). Risk factors for increased ED utilization in a multinational cohort of children with sickle cell disease. Acad. Emerg. Med..

[36] Field JJ, DeBaun MR (2009). Asthma and sickle cell disease: Two distinct diseases or part of the same process?. Hematol. Am. Soc. Hematol. Educ. Program.

[37] Patterson GD (2018). Recurrent acute chest syndrome in pediatric sickle cell disease: Clinical features and risk factors. J. Pediatr. Hematol. Oncol..

[38] Lopinto J (2021). Infectious aetiologies of severe acute chest syndrome in sickle-cell adult patients, combining conventional microbiological tests and respiratory multiplex PCR. Sci. Rep..

[39] Sprinkle RH (1986). Acute chest syndrome in children with sickle cell disease. A retrospective analysis of 100 hospitalized cases. Am. J. Pediatr. Hematol. Oncol..

[40] van Agtmael MA, Cheng JD, Nossent HC (1994). Acute chest syndrome in adult Afro-Caribbean patients with sickle cell disease. Analysis of 81 episodes among 53 patients. Arch. Intern. Med..

[41] Berger E (2009). Sickle cell disease in children: Differentiating osteomyelitis from vaso-occlusive crisis. Arch. Pediatr. Adolesc. Med..

[42] Kassim AA (2015). How I treat and manage strokes in sickle cell disease. Blood.

[43] Powell RW (1992). Acute splenic sequestration crisis in sickle cell disease: Early detection and treatment. J. Pediatr. Surg..

